# Pig Herds Free from Human Pathogenic *Yersinia enterocolitica*^1^

**DOI:** 10.3201/eid1312.070531

**Published:** 2007-12

**Authors:** Truls Nesbakken, Terje Iversen, Bjørn Lium

**Affiliations:** * Norwegian School of Veterinary Science, Oslo, Norway; † Nortura, Oslo, Norway; and ‡Animalia, Oslo, Norway

**Keywords:** *Yersinia Enterocolitica*, pigs, herd level, serology, culture, feces, public health, research

## Abstract

Pork products are a substantial source of human yersiniosis, a foodborne disease caused by *Yersinia enterocolitica*. Thus, the ability to eliminate this agent from pig herds would be an important step in producing human pathogen–free pork. Pig herds free from *Y. enterocolitica* O:3/biovar 4 have been established and maintained. According to serologic and cultural testing results, 15 of 16 specific pathogen–free herds were free from *Y. enterocolitica* O:3/biovar 4; this closed breeding pyramid has remained free from this organism since 1996. Pig herds free from human pathogenic *Y. enterocolitica* suggest that human pathogen–free herds could be attained to provide pork free from zoonotic agents.

## Abstract 2

Pig herds that provide pork free from zoonotic agents may be possible.

*Yersinia enterocolitica* is a major cause of foodborne disease in the industrialized world (*1**,**2*). The emergence of *Y. enterocolitica* O:3 and O:9 in Europe and Japan in the 1970s and in North America by the end of the 1980s has been characterized as an example of a global pandemic (*3*). Outbreaks of *Y. enterocolitica* O:3 have occurred among black US infants due to cross-contamination during household preparation of raw pork intestines (chitterlings) (*4**,**5*), and the main reservoir for *Y. enterocolitica* O:3 in Europe is the domestic pig population (*6*). A case–control study conducted by the US Centers for Disease Control and Prevention (CDC) and the Norwegian Institute of Public Health (NIPH) indicated pork products as a major source of yersiniosis in humans in Norway (*7*). As a result of this and other epidemiologic studies (*6**,**8**–**10*), improved slaughtering and dressing procedures of pigs (*11**,**12*) were implemented in Norwegian abattoirs in 1994. The decline in the incidence of human yersiniosis (*13*), which started in 1995, is most likely the result of these preventive measures. Among the Nordic countries, Denmark, Norway, and Sweden started to improve slaughter hygiene by implementing the plastic bag technique during 1990–1995; however, Finland did not implement this technique, which may have contributed to the higher level of human yersiniosis in this country than in the other Nordic countries (*14*).

During an outbreak in January and February 2006, 11 human cases of *Y. enterocolitica* O:9/biovar 2 infection were identified in Norway; 2 patients died and reactive arthritis developed in 1 (*15*). A case–control study and microbiologic findings indicated a processed pork product (julesylte; Christmas brawn) as the probable source. Another, smaller, family outbreak of yersiniosis occurred, caused by *Y. enterocolitica* O:3/biovar 4 in brawn and was registered in the outbreak database at NIPH in 2006 (*16*).

Most Norwegian pig production is organized in a closed breeding system in which primary nucleus-herd farms sell breeding animals to secondary multiplying-herd farms. These multiplying-herd farms sell breeding animals to conventional-herd farms (farrowing to finishing herds or young pig production). In turn, animals from young pig–production farms are sold to fattening-herd farms. These breeding pyramids are kept free from animal diseases such as sarcoptic mange, swine dysentery, and enzootic pneumonia. If successful elimination of human pathogenic *Y. enterocolitica* could be accomplished on the top levels of the breeding pyramids, prevalence of human pathogenic *Y. enterocolitica* might be lowered in the general pig population. Previously, Skjerve et al. (*17*) indicated that intervention at herd level is a possible strategy for maintenance of *Y. enterocolitica* O:3/biovar 4–free pig herds in Norway. Serologic analysis showed 182 (63.4%) of 287 herds to be positive for *Y. enterocolitica* O:3. Among the seropositive herds in this study, significantly fewer were mixed herds of piglets and fatteners (53.1%) than fattening herds (86%). Mixed herds represent a significant protective factor against infection with *Y. enterocolitica* O:3/biovar 4 because the herd is not supplemented by animals brought in from outside sources. Thus, reducing the herd prevalence of *Y. enterocolitica* O:3/biovar 4 may be possible by minimizing contact between infected and noninfected herds.

The ability to create pig herds free of human pathogenic *Y. enterocolitica* has been evaluated. We report that a specific pathogen–free (SPF) breeding pyramid with focus on animal disease can be established and maintained free from *Y. enterocolitica* O:3/biovar 4.

## Material and Methods

### Herds

In 1996, the first SPF nucleus herd (herd 1; 100 breeding sows) was established by hysterectomy, and the piglets were reared without contact with other pigs. In 1999, a second nucleus SPF herd (herd 2; 65 breeding sows) was established with gilts from herd 1. These 2 herds have been totally isolated from other herds, except for artificial insemination. Since 1997, 14 new SPF herds have been established with gilts from 1 or both of the above-mentioned SPF nucleus herds; each has been maintained as a closed herd (or supplemented with replacement gilts from 1 of the 2 SPF nucleus herds). Each of these 14 new SPF herds had an average of 60 animals (range 20–150). All SPF herds are housed, the water supply is potable, and pest control systems are established. Pets and wild animals cannot enter the pig house. The owner, herdsmen, veterinarians, and technicians must shower and change clothes before entering the pig housing. Many pig herds organized in the general closed breeding system have also implemented many of these preventive measures.

**Testing of pigs**

Previously, Nesbakken et al. (*18*) have shown that *Y. enterocolitica* O:3/biovar 4 can be detected in different age groups of pigs by 1) serologic testing of pigs at all ages from ≈100 days, including at slaughter when the pigs are 150–180 days old; and 2) bacteriologic examination of feces from pigs of all ages from 85 days until ≈135 days. In most instances, the testing of pigs in our study has been in accordance with the conclusions of Nesbakken et al (*18*).

### Collection of Blood Samples

After the original 54 samples were tested in 1996, blood samples from 30–60 pigs in herd 1 were tested for antibodies against *Y. enterocolitica* O:3 every year from 1998 through 2007, and samples from 30 pigs in herd 2 were tested each year from 2001 through 2006. Periodically, from 2002 through 2007, blood samples from 19–60 pigs from the 14 secondary SPF herds were tested (Table). Most blood samples were collected from 4- to 6-month-old fatteners or gilts. Through 2001, some samples from pigs in the 2 nucleus herds were from sows. In total, blood samples from 1,083 pigs from 16 different herds were tested for antibodies against *Y. enterocolitica* O:3.

**Table:** Antibodies against *Yersinia enterocolitica* O:3 in blood samples and culture of feces from pigs in a closed system of 16 SPF herds in Norway*

Herd no. (year established)	Serologic testing (1996–2007), no. pos/no. tested	Culture (2005–2006), no. pos./no. tested
1 (1996)	10/397†	0/20
2 (1999)	0/150	0/20
3 (1997)	1/61	0/21
4 (1997)	0/19	0/20
5 (1998)	0/30	NA‡
6 (1999)	0/34	0/20
7 (1999)	0/20	0/20
8 (2000)	0/60	0/20
9 (2001)	0/30	NA‡
10 (2002)	1/61	0/20
11 (2002)	0/20	0/20
12 (2003)	0/30	0/22
13 (2003)	0/51	0/18
14 (2004)	15/30	11/24§
15 (2004)	0/50	0/23
16 (2004)	0/30	0/20

*SPF, specific pathogen–free. Herds 1 and 2 are nucleus herds. Herds 3–16 were established with gilts from 1 or both of the nucleus herds. A basic cut-off of optical density of 20% was used to maximize the specificity of the ELISA. †During the first 5 years, 10 of 174 blood samples from pigs in herd 1 had a low level of antibodies against *Y. enterocolitica* O:3 (OD >20 but <31). None of the 223 blood samples taken from pigs in this herd from 2002 through 2007 was positive. The low-positive reactions from pigs in herd 1 might have been the result of nonspecific reactions because a few of these samples were from old sows, which might have more serologic interference. ‡NA, not applicable; no culture of feces. §Positive for *Y. enterocolitica* O:3/biovar 4.

### Collection of Fecal Samples

Each herd was sampled once. In total, 286 samples were collected from 18–24 animals from each of 4 herds in 2005 and 10 herds in 2006 (Table). Fecal samples were not collected from herds 5 (the owner did not give permission) and 9 (no longer registered as an SPF herd since 2006). Fecal samples weighed 0.1–36.8 g. The average amounts per herd tested varied from an average of 0.8 g (range 0.1–3.3 g) to an average of 23 g (range 8–31 g). The fecal samples were aseptically collected from the rectum of the pigs (86–150 days of age) by use of a clean plastic glove.

### Serologic Methods

Serum samples were analyzed for antibodies against *Y. enterocolitica* O:3 by using an indirect pig immunoglobulin lipopolysaccharide ELISA (*19*) at the Danish Veterinary Institute, Technical University of Denmark, Copenhagen. A basic cut-off of optical density (OD) 20% was used to maximize the specificity of the ELISA.

### Isolation and Characterization of *Y. enterocolitica*

*Y. enterocolitica* were cultured and isolated according to the International Organization for Standardization (*20*) with modifications (*21**,**22*). Colonies characteristic for *Yersinia* were confirmed biochemically, first by selecting only lactose-negative, urease-positive colonies and later with Vitek (BioMerieux Limited, Marcy l’Etoile, France) by using the revised biogrouping scheme for *Y. enterocolitica* (*23*) as a key, and serologically for O:3 and O:9 reactivity (63501 and 63502; Sanofi Diagnostics-Pasteur, Marnes la Coquette, France).

## Results and Discussion

The serologic and the bacteriologic results showed a low rate of exposure to *Y. enterocolitica* O:3/biovar 4 in the pigs from the closed SPF herds (Table). During the first 5 years, 10 of 174 blood samples from pigs in herd 1 had low levels of antibodies against *Y. enterocolitica* O:3; however, because some of these pigs were old sows, the low titers (OD >20% but <31%) are consistent with past exposure to the organism or nonspecific cross-reaction rather than active infection. Bowman et al. (*24*) report that gestating sows had the second highest prevalence of human pathogenic *Y. enterocolitica* among the different age categories at herd level; *Y. enterocolitica* was never detected in the farrowing sows. Gürtler et al. (*25*) did not detect human pathogenic *Y. enterocolitica* among sows. However, according to these 2 reports, the sows were investigated by culture and not by serologic testing (*24**,**25*). In the past 5 years (2002–2007), none of the 223 blood samples taken from pigs in this herd has been positive for *Y. enterocolitica.* Although some of the blood samples from the 2 nucleus herds were from old sows, most were from fattening pigs at slaughter. If nucleus herd 1 had been truly positive, pigs purchased from this herd would probably have infected the other herds because this herd was at the top of the breeding pyramid. In herds 3 and 10, 1 of 61 animals was positive. When a herd has a history of infection with *Y. enterocolitica* O:3/biovar 4, antibodies are widely distributed among the animals (*17**,**18*). Accordingly, it is not likely that herds 1, 3, and 10 were infected by *Y. enterocolitica* O:3/biovar 4. The specificity of the serologic ELISA used is not fully known; false positives might appear. Only 1 of the 16 herds examined (herd 14) was classified as serologically positive for antibodies against *Y. enterocolitica* O:3. Among the 30 animals tested, 15 were positive (OD average 39%; range 0%–109%). This herd was also the only one that was positive for *Y. enterocolitica* O:3/biovar 4 according to culture result. The isolation method used in our study has proven to be sensitive for isolation of *Y. enterocolitica* O:3/biovar 4 even when the fecal samples are small (*18**,**21*). On the basis of intestinal tract content samples (n = 120), there was no statistical difference between the isolation method used in our study and the BUGS’n BEADS (Genpoint, Oslo, Norway) detection method (PCR) for virulent *Y. enterocolitica* (*21*).

According to serologic testing results, 15 of the 16 SPF herds examined were free from *Y. enterocolitica* O:3/biovar 4. The first basic nucleus herd at the top of this breeding pyramid has remained free from this pathogenic variant since the herd’s establishment in 1996. A total of 13 herds were confirmed negative for *Y. enterocolitica* O:3/biovar 4 by culture of feces. Broadly, these findings show that clusters of pig herds free from *Y. enterocolitica* O:3/biovar 4 can be established and kept free from this human pathogenic variant for many years. Christensen (*26*) also documented a low level of human pathogenic *Y. enterocolitica* in 4 SPF herds examined by tonsil swabs in Denmark during 1978–1979. From 99 pigs he found only 1 isolate of *Y. enterocolitica* serovar O:3/biovar 4.

The low prevalence of human pathogenic *Y. enterocolitica* observed in the herds’ immediate environment (e.g., water, rodents, flies) by Pilon et al. (*27*) suggests that the environment does not represent the main source of contamination of pigs by human pathogenic *Y. enterocolitica.* Rather, transmission is more likely from other infected pigs. Thus, mixed herds in closed health and breeding pyramids represent an important barrier against infection with *Y. enterocolitica* O:3/biovar 4. Reduction in prevalence of human pathogenic *Y. enterocolitica* at the top levels of the health and breeding pyramids may also reduce the prevalence of *Y. enterocolitica* O:3/biovar 4 in the general pig population. The meat industry could then categorize herds by serologic or bacteriologic methods and use these results in its strategy to reduce the risks for consumers. Serologic testing is preferable to bacteriologic methods on the basis of practicality, time-saving aspects, and costs. If human pathogenic *Y. enterocolitica–*free segments of the pig population could be established, preharvest risk management might be possible by using serologic methods to categorize herds. If this experience is used in the general health and breeding pyramids of pig herds, the Norwegian meat industry could provide pork from pigs raised in herds free from human pathogenic *Y. enterocolitica*, which might be the starting point for providing human pathogen–free (HPF) pork on the market. The following facts should be considered in discussions of the possibility of establishing HPF herds: 1) <0.1% of the pigs in Norway harbor *Salmonella* (*28*); 2) the most recent case of *Trichinella* infection in pigs was in 1994 (*28*); 3) 2.6% of 1,605 pigs from 321 herds had antibodies against *Toxoplasma gondii* (*29*), and only 1.3% of the mixed herds had antibodies against *T. gondii* according to the data on which this article is based; and 4) ≈100% of the pigs harbor *Campylobacter* spp. (*21*).

Closed SPF pig herds are probably nearly free from *Salmonella, Trichinella,* *T. gondii,* and, according to our findings, even human pathogenic *Y. enterocolitica*. Freedom from *Campylobacter* spp. in pigs is probably impossible. However, blast chilling after the slaughtering process seems to reduce the number of *Campylobacter* spp. ≈100% (*30*; Nesbakken et al., unpub. data). Thus, in the future, pork from Norwegian SPF pig herds and even mixed herds in closed breeding pyramids might be marketed as HPF.

Another aspect to consider is the environment. Usually manure from pig farms is spread in fields and may contaminate wild animals, lakes, and rivers. Drinking water may thereby be contaminated with pathogenic *Y. enterocolitica.* This contamination has a human health aspect because one of the risk factors for human yersiniosis might be drinking water that has not been disinfected (*7*). Thus, in addition to their public health benefits, human pathogenic *Y. enterocolitica–*free herds might have a positive environmental effect.
